# Supplementary material for: Antiviral Mx proteins have an ancient origin and widespread distribution among eukaryotes
Source: bioRxiv. 2024 Aug 17:2024.08.06.606855. Originally published 2024 Aug 6. Preprint. [Version 2] doi: 10.1101/2024.08.06.606855 (PMC11326297; doi:10.1101/2024.08.06.606855)
Supplement: Supplement 2 [file NIHPP2024.08.06.606855v2-supplement-2.pdf]

bioRxiv preprint doi: https://doi.org/10.1101/2024.08.06.606855; this version posted August 17, 2024. The copyright holder for this preprint (which was not certified by peer review) is the author/funder, who has granted bioRxiv a license to display the preprint in perpetuity. It is made available under aCC-BY 4.0 International license.

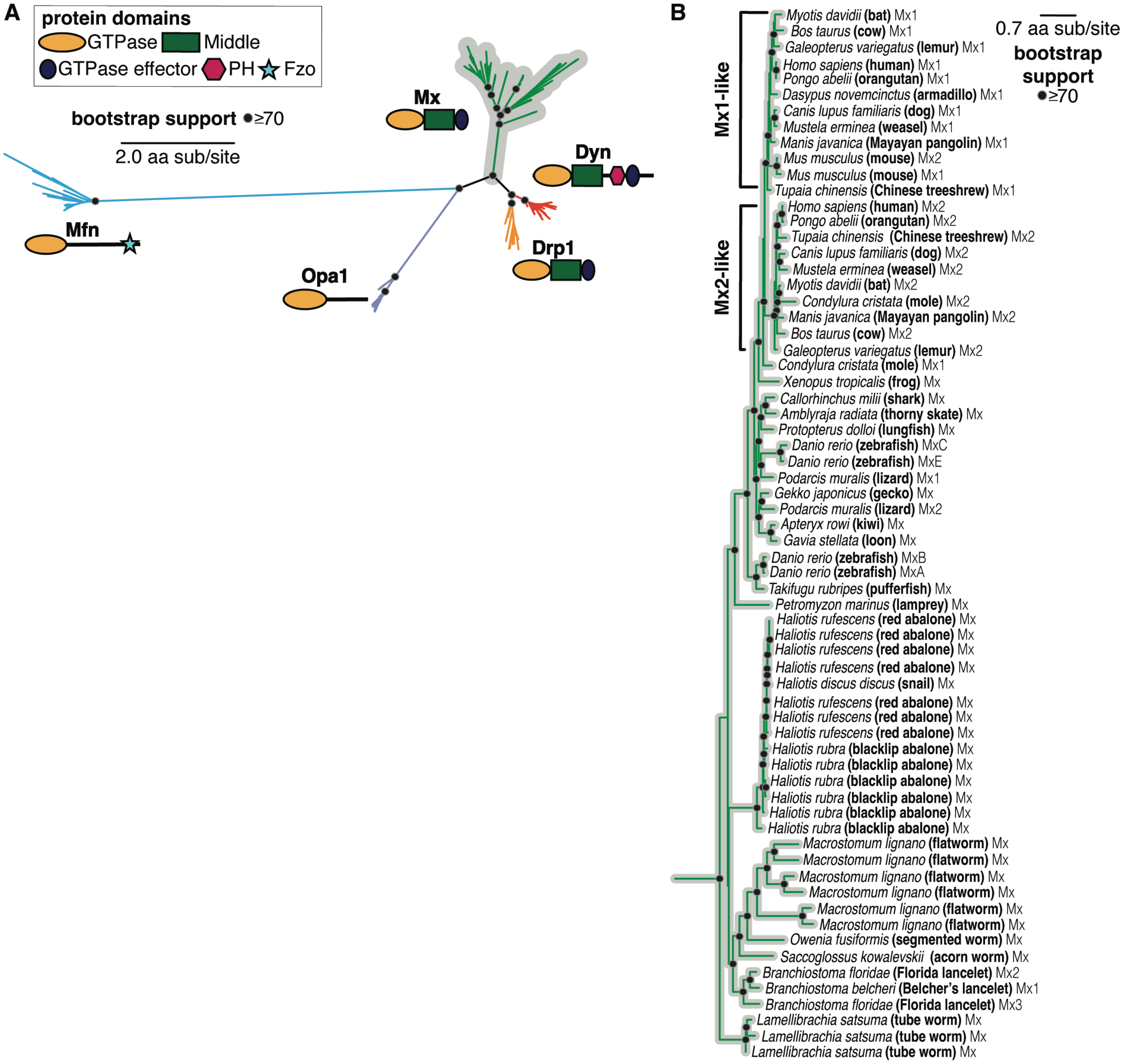

Supplementary Figure 1. The evolutionary origin of Mx in animals predates the interferon signaling network. (A) Phylogenetic analysis of Dynamin-superfamily proteins (DSPs) based on their common GTPase domain in representative Holozoa (animals and their closest single-celled relatives) reveals five distinct DSP clades, consistent with Figure 1A. We used MAFFT to generate the alignment and IQ-Tree (112,113) to build the phylogeny. Black dots indicate nodes with UltraFast bootstrap support greater than 70% based on IQ-Tree analyses (see methods); a scale bar indicates the level of amino acid divergence. (B) Phylogenetic analysis of Mx-like proteins in animal species, consistent with Figure 1D.

bioRxiv preprint doi: https://doi.org/10.1101/2024.08.06.606855; this version posted August 17, 2024. The copyright holder for this preprint (which was not certified by peer review) is the author/funder, who has granted bioRxiv a license to display the preprint in perpetuity. It is made available under aCC-BY 4.0 International license.

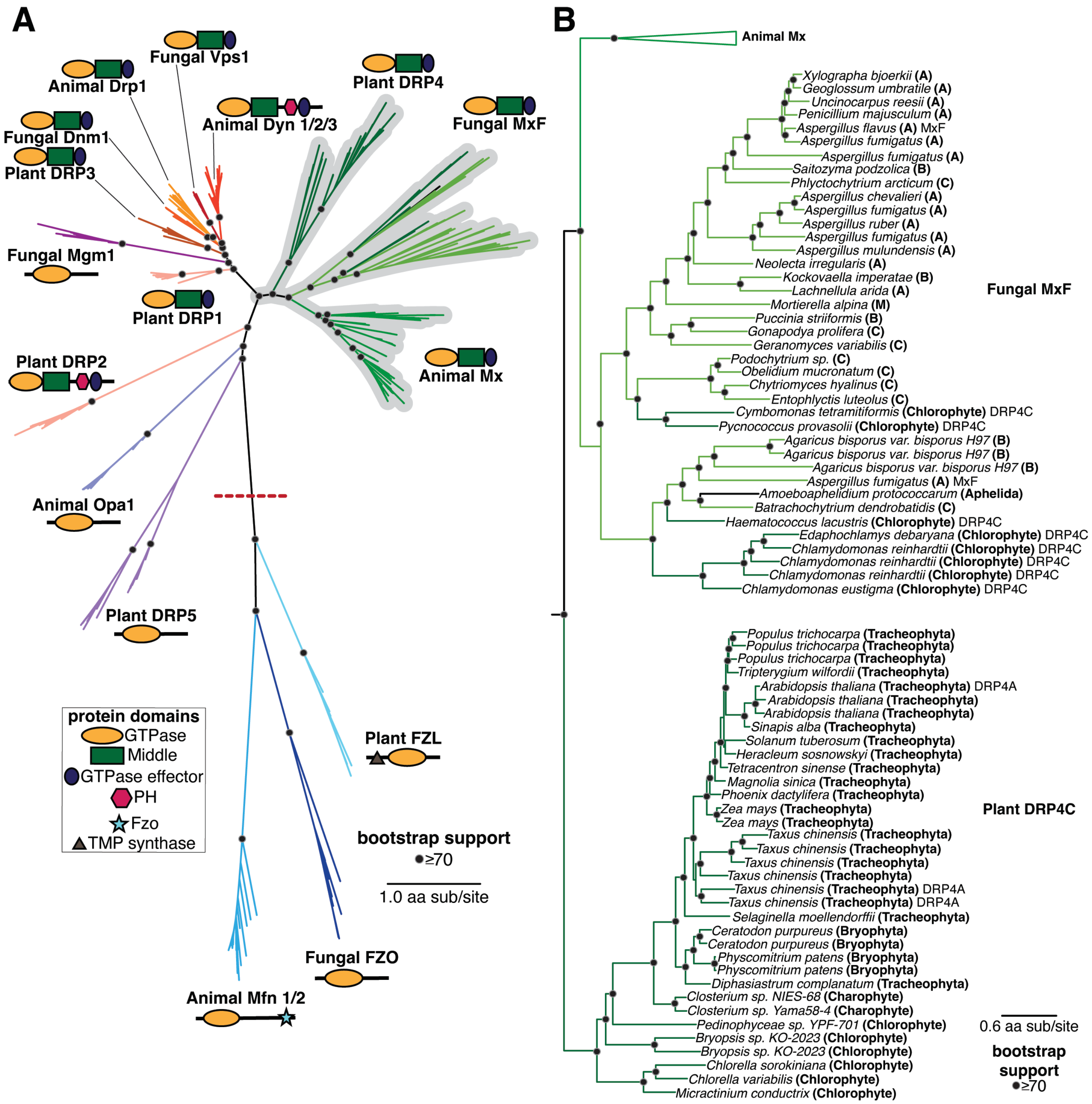

Supplementary Figure 2. Animal DSP clades, including Mx proteins, in fungal and plant genomes. (A) Phylogenetic analysis of animal, fungal, and plant DSPs based on their common GTPase domain reveals broad groupings consistent with FastTree analysis in Figure 2A. We used MAFFT to generate the alignment and IQ-Tree (112,113) to build the phylogeny. Black dots indicate nodes with UltraFast bootstrap support greater than 70% based on IQ-Tree analyses (see methods); a scale bar indicates the level of amino acid divergence. **(B)** Phylogenetic analysis of animal Mx, fungal MxF (Mx-like proteins from Fungi), and plant DRP4 proteins, consistent with Figure 2C.

bioRxiv preprint doi: https://doi.org/10.1101/2024.08.06.606855; this version posted August 17, 2024. The copyright holder for this preprint (which was not certified by peer review) is the author/funder, who has granted bioRxiv a license to display the preprint in perpetuity. It is made available under aCC-BY 4.0 International license.

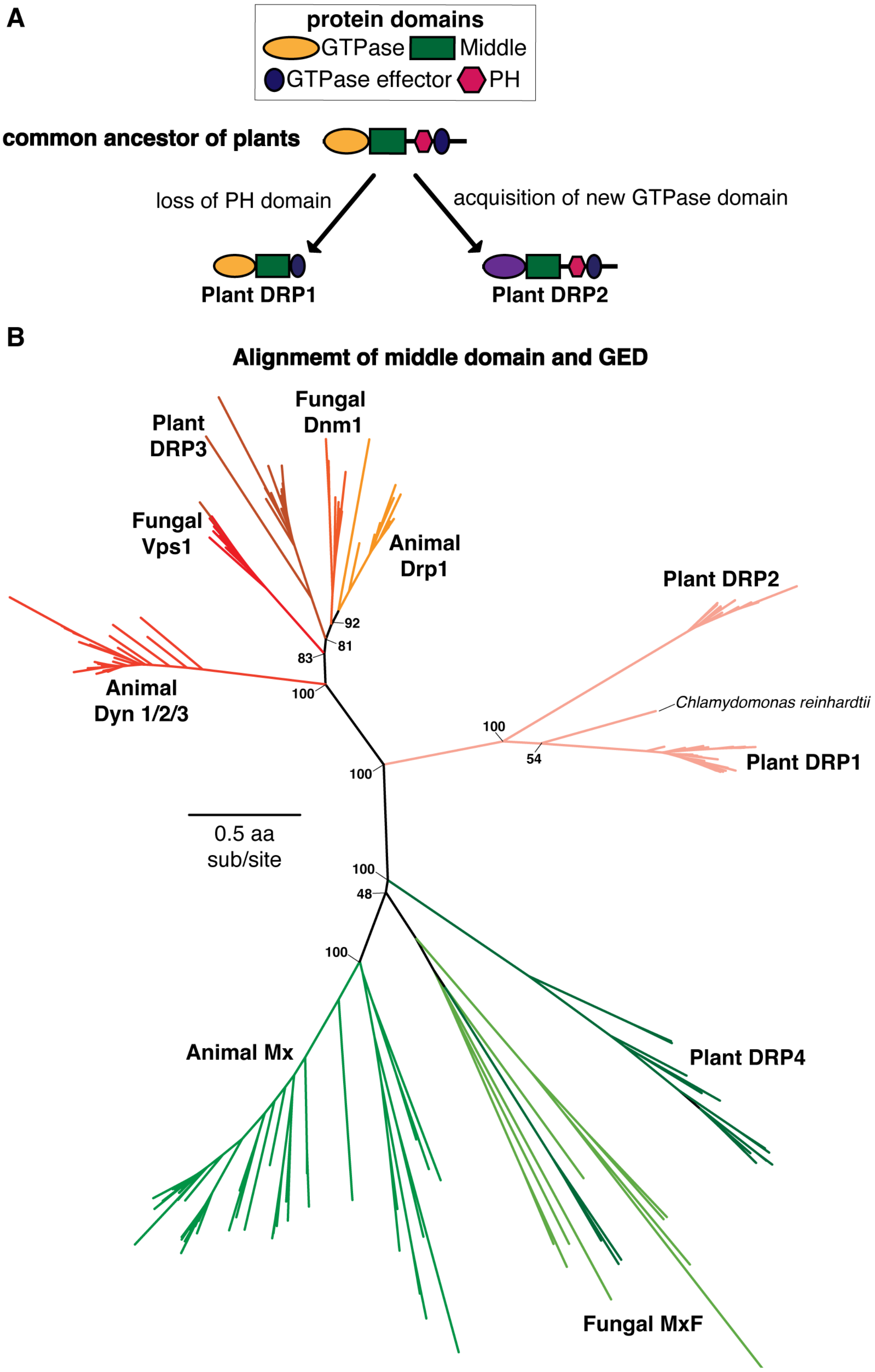

Supplementary Figure 3. Evolutionary analyses of Plant DRP1 and DRP2 proteins. (A) A proposed model of Plant DRP1 and DRP2 evolution. The plant DRP1/DRP2 ancestor contained GTPase, Middle, GED, and PH domains. This ancestor was duplicated into DRP1, which lost its PH domains, and DRP2, which retained the PH domain but acquired a divergent GTPase domain via recombination. **(B)** Phylogenetic analysis of Plant, Animal, and Fungi DSP Middle and GED domains reveals that Plant DRP1 and DRP2 are much more closely related in the Middle-GED phylogeny than in the GTPase phylogeny (Figure 2). Although this does not clarify where the Plant DRP2 GTPase domain arose from (but see Figure 3), the rest of the protein appears to be derived from a common Plant DRP1/2 ancestor.

bioRxiv preprint doi: https://doi.org/10.1101/2024.08.06.606855; this version posted August 17, 2024. The copyright holder for this preprint (which was not certified by peer review) is the author/funder, who has granted bioRxiv a license to display the preprint in perpetuity. It is made available under aCC-BY 4.0 International license.

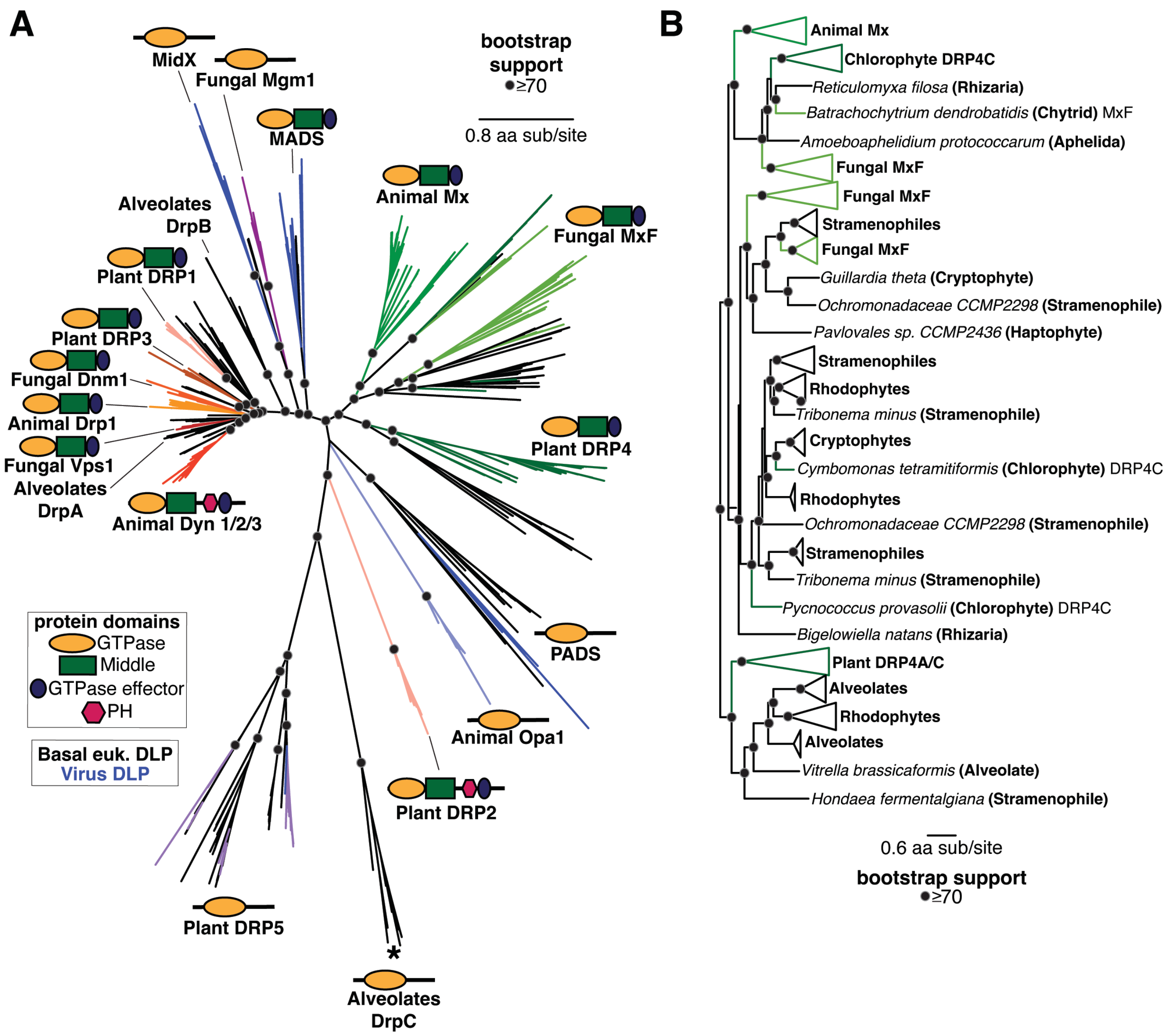

Supplementary Figure 4. Phylogenetic analysis of DSPs in eukaryotes reveals an ancient Mx lineage. (A) Phylogenetic analysis of all eukaryotic DSPs (except for the Mfn/FZO, FZB, and FZL clades) reveals an ancient Dyn/Drp clade that includes Animal Drp, Plant DRP3, and Fungal Vsp1 along with fungal Dnm1, alveolate DrpA and DrpB, Plant DRP1, and DSPs from several basal branching eukaryotes (shown with black branches). The tree topology is consistent with FastTree analysis in Figure 3A. We used MAFFT to generate the alignment and IQ-Tree (112,113) to build the phylogeny. Black dots indicate nodes with UltraFast bootstrap support greater than 70% based on IQ-Tree analyses (see methods); a scale bar indicates the level of amino acid divergence. **(B)** Phylogenetic analysis of three deeply branching lineages of Mx-like proteins in eukaryotes, compared to Figure 3B
